# Supplementary material for: Nanobody-based RFP-dependent Cre recombinase for selective anterograde tracing in RFP-expressing transgenic animals
Source: Commun Biol. 2022 Sep 16;5:979. doi: 10.1038/s42003-022-03944-2 (PMC9481622; doi:10.1038/s42003-022-03944-2)
Supplement: Supplementary file 2 — Supplementary information [file 42003_2022_3944_MOESM2_ESM.pdf]

Supplementary Information

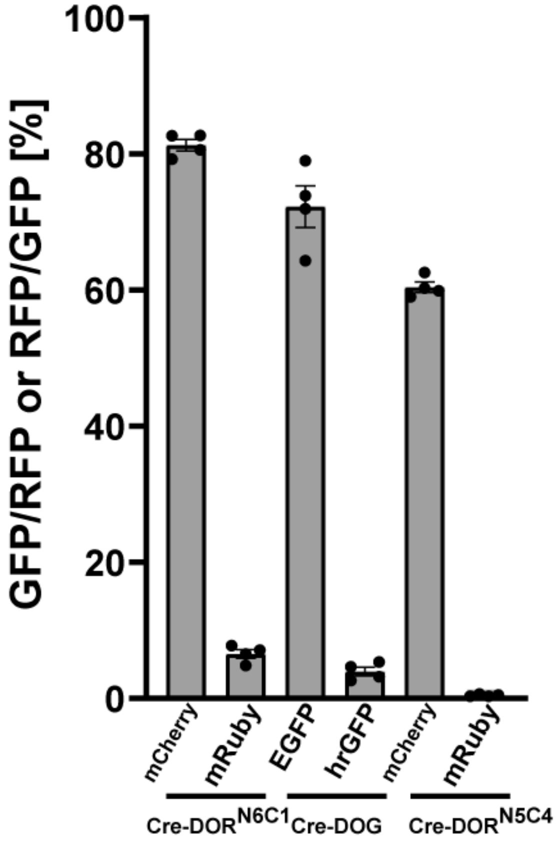

Supplementary Figure 1. Benchmarking of Cre-DOR with Cre-DOG.

Quantification of cell counts in transfected HEK293 cells to assess Cre-DOR^{N6C1}, Cre-DOG, and Cre-DOR^{N5C4} efficiency. Four kinds of plasmids (NCre-MBP, CCre-MBP, FLEX-H2B-GFP or FLEX-H2B-RFP, and target RFPs or GFPs) were transfected in HEK293 cells to assess Cre-DOR or Cre-DOG recombination efficiency. Data are means ± SEM (n = 4).

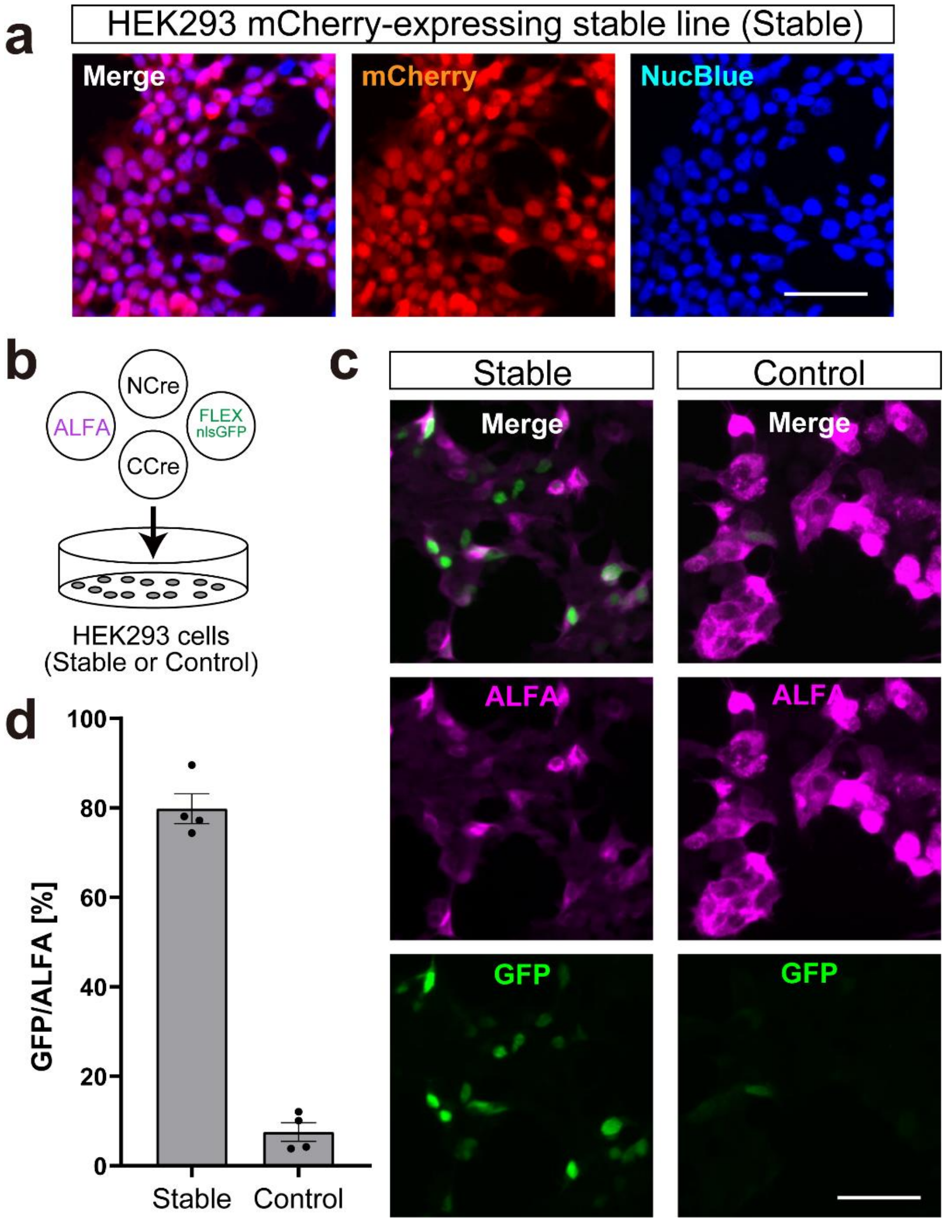

Supplementary Figure 2. Assessment of Cre-DOR using an mCherry-expressing stable line.

a, Confirmation of mCherry expression in the mCherry-expressing stable line (AST-1320). ***b,*** Schematic illustration of Cre-DOR transfection in HEK293 cells. Four kinds of plasmids (NCre-MBP, CCre-MBP, FLEX-nlsGFP, and a transfection marker, ALFA) were transfected in HEK293 cells to assess Cre-DOR recombination efficiency. ***c,*** Fluorescent images of reporter nlsGFP expression in transfected HEK293 cells to assess Cre-DOR efficiency. ***d,*** Quantification of cell counts in transfected HEK293 cells to assess Cre-DOR (N-Cre-MBP6 and C-Cre-MBP1) efficiency. Data are means ± SEM (n =4 each). Scale bar = 50 μm.

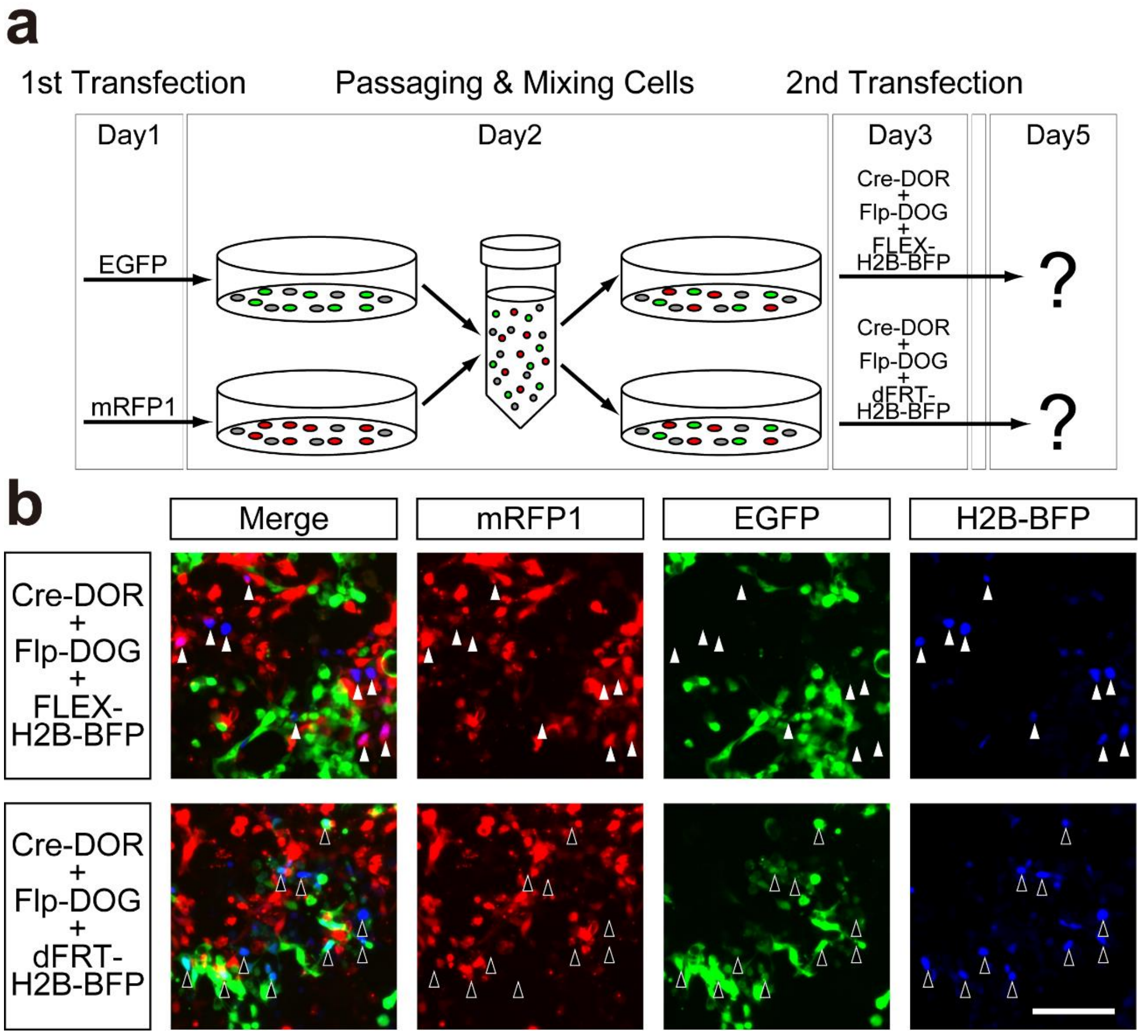

Supplementary Figure 3. Orthogonality of recombination activity between Cre-DOR and Flp-DOG.

a, Schematic presentation of experimental protocols. Day1; EGFP or mRFP1 was transfected into HEK293 cells. Day2; Transfected cells were passaged and mixed in the same dishes. Day3; Four kinds of plasmids including N-Cre-MBP6, C-Cre-MBP1, Flp recombinase dependent on GFP (Flp-DOG) and FLEX-H2B-BFP or dFRT-H2B-BFP were co-transfected into the HEK293 cells. Day5; Recombinase activities were detected as H2B-BFP expression induced by FLEX or dFRT switching. ***b***, Fluorescent images of reporter H2B-BFP expression in transfected HEK293 cells to assess orthogonality of Cre-DOR^{N6C1} to Flp-DOG. Cre-DOR induced mRFP1-dependent expression of FLEX-H2B-BFP (white arrowheads), while Flp-DOG induced GFP-dependent expression of dFRT-BFP (black arrowheads). The fluorescent signal of BFP was directly observed without immunostaining. The Flp-DOG plasmid was purchased from Addgene (#75469). Scale bar = 100 µm.

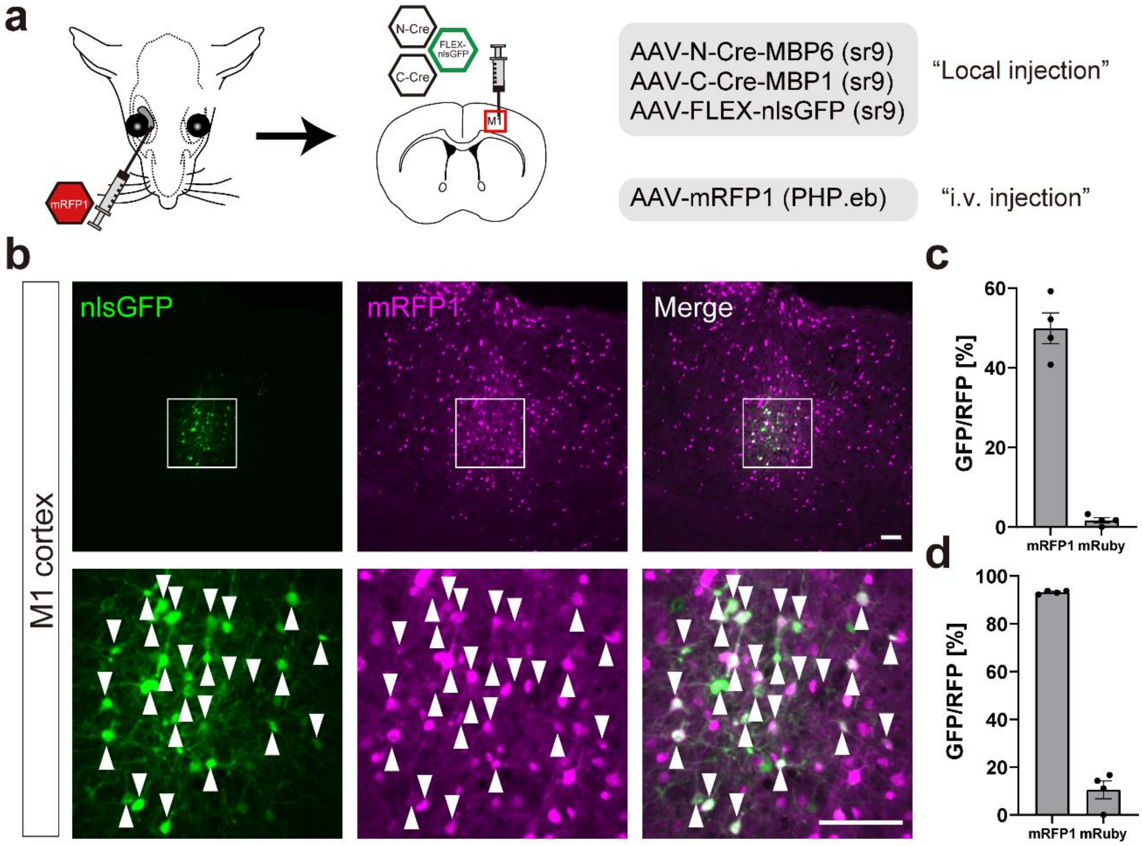

Supplementary Figure 4 . Functional assay of Cre-DOR^{N6C1} *in vivo* using i.v. injection of target RFP vectors.

a, Injection schema of the Cre-DOR^{N6C1} test with mRFP1 in wild-type mice. Target RFP vectors were injected intravenously. Three kinds of virus (NCre-MBP6, CCre-MBP1, and FLEX-nlsGFP) were injected in the M1 cortex at the same time. ***b,*** Fluorescent images of the M1 cortex. Scale bar = 100 μm. ***c,*** Quantification of cell counts to assess the efficiency of Cre-DOR^{N6C1} (n = 4 each). ***d,*** Quantification of cell counts to assess the fidelity of Cre-DOR^{N6C1} (n = 4 each). Data are means ± SEM. The illustration of retro-orbital injection in ***a*** was previously created for (Inutsuka, 2020)[1] and modified for this figure. (Inutsuka, 2020) is an open access article under a Creative Commons Attribution 4.0 International License (https://creativecommons.org/licenses/by/4.0/).

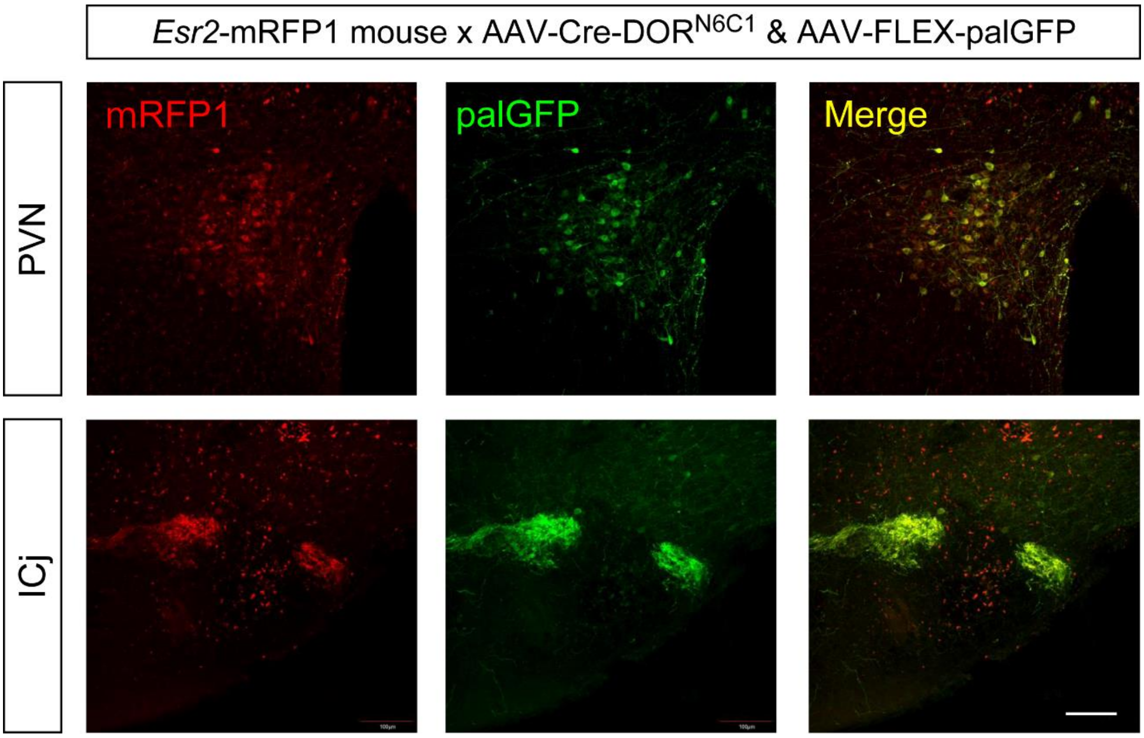

Supplementary Figure 5. Selective expression of palGFP induced by Cre-DOR^{N6C1} in *Esr2*-mRFP1 transgenic mice.

1 µl of a mixture of AAV9-EF1α-NCre-MBP6-WPRE (6 x 10^{12} vg/ml), AAV9-EF1α-CCre-MBP1-WPRE (6 x 10^{12} vg/ml) and AAV9-CAG-FLEX-palGFP-WPRE (6 x 10^{12} vg/ml) was injected in the paraventricular nucleus (PVN) and islands of Calleja (ICj) of an old *Esr2*-mRFP1 transgenic mouse. Four weeks after injection, the mouse was sacrificed for immunohistochemistry and brain slices were stained with anti-GFP and anti-mRFP1. Scale bar = 100 µm.

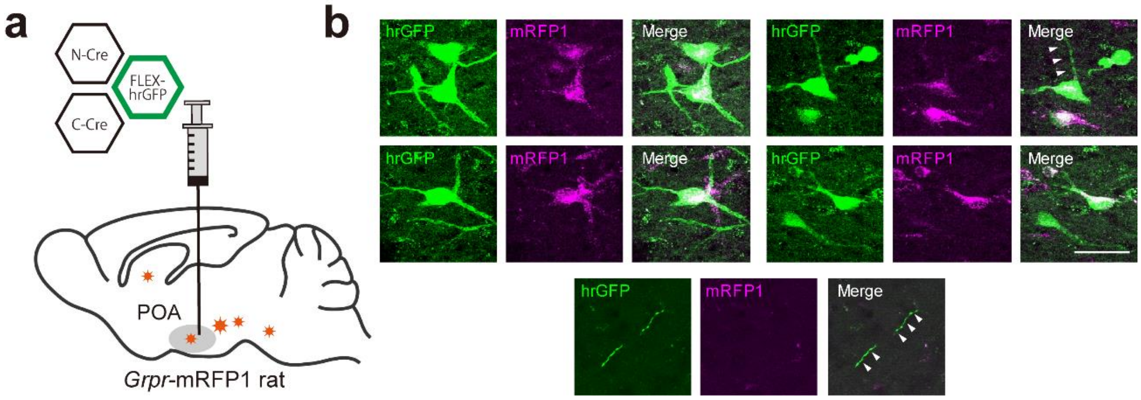

Supplementary Figure 6. Sparse labeling of mRFP-expressing neurons in *Grpr*-mRFP1 rats using Cre-DOR.

a, Schematic of Cre-DOR^{N6C1} virus injection in mRFP-expressing transgenic rats. Three kinds of virus (NCre-MBP6, CCre-MBP1, and FLEX-hrGFP) were injected in the preoptic area of Grpr-mRFP1 rats. ***b***, Fluorescent images of injected Grpr-mRFP1 rats. Specific expression of hrGFP was observed in mRFP1-expressing neurons. Scale bar = 50 µm.

References

1. Inutsuka A, Kimizuka N, Takanohashi N, Yakabu H, Onaka T. Visualization of a blue light transmission area in living animals using light-induced nuclear translocation of fluorescent proteins. *Biochem Biophys Res Commun* **522**, 138-143 (2020).
