# Supplementary material for: Nanobody-based RFP-dependent Cre recombinase for selective anterograde tracing in RFP-expressing transgenic animals
Source: Commun Biol. 2022 Sep 16;5:979. doi: 10.1038/s42003-022-03944-2 (PMC9481622; doi:10.1038/s42003-022-03944-2)
Supplement: Supplementary file 5 — Reporting Summary [file 42003_2022_3944_MOESM5_ESM.pdf]

nature portfolio

Corresponding author(s): Ayumu Inutsuka

Last updated by author(s): Jul 29, 2022

# Reporting Summary

Nature Portfolio wishes to improve the reproducibility of the work that we publish. This form provides structure for consistency and transparency in reporting. For further information on Nature Portfolio policies, see our Editorial Policies and the Editorial Policy Checklist.

## Statistics

For all statistical analyses, confirm that the following items are present in the figure legend, table legend, main text, or Methods section.

| n/a | Confirmed | |
|---|---|---|
| ☐ | ☒ | The exact sample size ($n$) for each experimental group/condition, given as a discrete number and unit of measurement |
| ☐ | ☒ | A statement on whether measurements were taken from distinct samples or whether the same sample was measured repeatedly |
| ☐ | ☒ | The statistical test(s) used AND whether they are one- or two-sided<br>*Only common tests should be described solely by name; describe more complex techniques in the Methods section.* |
| ☐ | ☒ | A description of all covariates tested |
| ☐ | ☒ | A description of any assumptions or corrections, such as tests of normality and adjustment for multiple comparisons |
| ☐ | ☒ | A full description of the statistical parameters including central tendency (e.g. means) or other basic estimates (e.g. regression coefficient) AND variation (e.g. standard deviation) or associated estimates of uncertainty (e.g. confidence intervals) |
| ☐ | ☒ | For null hypothesis testing, the test statistic (e.g. $F$, $t$, $r$) with confidence intervals, effect sizes, degrees of freedom and $P$ value noted<br>*Give P values as exact values whenever suitable.* |
| ☒ | ☐ | For Bayesian analysis, information on the choice of priors and Markov chain Monte Carlo settings |
| ☒ | ☐ | For hierarchical and complex designs, identification of the appropriate level for tests and full reporting of outcomes |
| ☒ | ☐ | Estimates of effect sizes (e.g. Cohen's $d$, Pearson's $r$), indicating how they were calculated |

*Our web collection on statistics for biologists contains articles on many of the points above.*

## Software and code

Policy information about availability of computer code

| | |
|---|---|
| Data collection | SparkControl magellan 2.2 (luciferase assay), MetaVue ver. 7.1.01.161 (fluorescence imaging), BZ-X Viewer 01.03.01.01 (fluorescence imaging) |
| Data analysis | GraphPad Prism 9, Image J 1.49v |

For manuscripts utilizing custom algorithms or software that are central to the research but not yet described in published literature, software must be made available to editors and reviewers. We strongly encourage code deposition in a community repository (e.g. GitHub). See the Nature Portfolio guidelines for submitting code & software for further information.

## Data

Policy information about availability of data

All manuscripts must include a data availability statement. This statement should provide the following information, where applicable:

- Accession codes, unique identifiers, or web links for publicly available datasets
- A description of any restrictions on data availability
- For clinical datasets or third party data, please ensure that the statement adheres to our policy

All the data are available from the corresponding author upon suitable request.

# Field-specific reporting

Please select the one below that is the best fit for your research. If you are not sure, read the appropriate sections before making your selection.

☒ Life sciences ☐ Behavioural & social sciences ☐ Ecological, evolutionary & environmental sciences

For a reference copy of the document with all sections, see nature.com/documents/nr-reporting-summary-flat.pdf

# Life sciences study design

All studies must disclose on these points even when the disclosure is negative.

| | |
|---|---|
| Sample size | Sample sizes adequate for statistical tests were determined based on previous experiments from our laboratory and others. |
| Data exclusions | No data were excluded from the analyses. |
| Replication | All attempts of replication were successful. |
| Randomization | Animals were randomly assigned to each experimental group. |
| Blinding | For manual cell counting, the investigators were blinded to group allocation. |

# Reporting for specific materials, systems and methods

We require information from authors about some types of materials, experimental systems and methods used in many studies. Here, indicate whether each material, system or method listed is relevant to your study. If you are not sure if a list item applies to your research, read the appropriate section before selecting a response.

## Materials & experimental systems

| n/a | Involved in the study | |
|---|---|---|
| ☐ | ☒ | Antibodies |
| ☐ | ☒ | Eukaryotic cell lines |
| ☒ | ☐ | Palaeontology and archaeology |
| ☐ | ☒ | Animals and other organisms |
| ☒ | ☐ | Human research participants |
| ☒ | ☐ | Clinical data |
| ☒ | ☐ | Dual use research of concern |

## Methods

| n/a | Involved in the study | |
|---|---|---|
| ☒ | ☐ | ChIP-seq |
| ☒ | ☐ | Flow cytometry |
| ☒ | ☐ | MRI-based neuroimaging |

## Antibodies

| | |
|---|---|
| Antibodies used | anti-GFP (RRID: AB_591819, Medical & Biological Laboratories)<br>anti-GFP (RRID: AB_1537403, Rockland Immunochemicals)<br>anti-GFP (RRID: AB_2314545, Nacalai tesque)<br>anti-DsRed (RRID: AB10013483, Takara Bio)<br>anti-ALFA (NanoTag Biotechnologies, N1580)<br>Alexa Fluor 488 goat anti-rabbit IgG (A11034; Thermo Fisher Scientific)<br>Alexa Fluor 488 donkey anti-rat IgG (A21208; Thermo Fisher Scientific)<br>Alexa Fluor 488 goat anti-chicken IgY (103-545-155; Jackson ImmunoResearch Laboratories)<br>Alexa Fluor 555 goat anti-rabbit IgG (A21428; Jackson ImmunoResearch Laboratories)<br>Alexa Fluor 647 donkey anti-rabbit IgG (ab150075; abcam) |
| Validation | All the primary antibodies used in the manuscript were identified and validated by RRIDs (Research Resource Identifiers) except anti-ALFA antibody (NanoTag Biotechnologies, N1580).<br>Validation statements of the secondary antibodies are available from manufacturers: |

## Eukaryotic cell lines

Policy information about cell lines

| | |
|---|---|
| Cell line source(s) | AAV-293 cells (purchased from Agilent technologies). AAV-293 cells are derived from the commonly used HEK293 cell line, but produce higher viral titers. An mCherry-expressing HEK293 cell line was purchased from Applied StemCell (AST-1320). |
| Authentication | None of the cell lines used were authenticated. |

| | |
|---|---|
| Mycoplasma contamination | The cell line was not tested for mycoplasma contamination. |
| Commonly misidentified lines (See ICLAC register) | No commonly misidentified lines were used in our laboratory. |

## Animals and other organisms

Policy information about studies involving animals; ARRIVE guidelines recommended for reporting animal research

| | |
|---|---|
| Laboratory animals | We used 10-week-old maleC57BL/6J mice, 12-14-week-old male and female Esr2-mRFP1 transgenic mice, and 10–15-week-old female Grpr-mRFP1 transgenic rats for virus injections. C57BL/6J mice were purchased from Charles River Laboratories Japan. Esr2-mRFP1 transgenic mice were generated by Dr. Hirotaka Sakamoto and their validation was published (Sagoshi et al., 2020-Neuroscience). Grpr-mRFP1 transgenic rats were also generated by Dr. Hirotaka Sakamoto and their validation was published (Takanami et al., 2021-Proc Natl Acad Sci U S A ). |
| Wild animals | n/a |
| Field-collected samples | n/a |
| Ethics oversight | All experimental procedures with mice were approved by the Institutional Animal Experiment Committee of Jichi Medical University or the Animal Care and Use Committee, Okayama University. |

Note that full information on the approval of the study protocol must also be provided in the manuscript.
